# Physical Activity to Improve Lung Cancer Survival: Promising Evidence

**DOI:** 10.1093/jncics/pkac011

**Published:** 2022-02-14

**Authors:** Christine M Friedenreich, Lin Yang

**Affiliations:** 1 Department of Cancer Epidemiology and Prevention Research, Cancer Care Alberta, Alberta Health Services, Holy Cross Center, Calgary, Canada; 2 Department of Oncology and Community Health Sciences, Cumming School of Medicine, University of Calgary, Calgary, Canada

We read with great interest the pooled analysis of 11 cohort studies by Yang et al. (1) in this issue of the Journal that examined how prediagnosis leisure-time (recreational) physical activity is associated with survival after lung cancer. The authors found a statistically significant 7% lower hazard of all-cause mortality among study participants who achieved 8.3 MET -h/wk or more of recreational physical activity compared with participants who were inactive. A stronger protective effect was found for those participants with localized cancer for whom a 20% lower mortality was observed for both all-cause and lung cancer–specific mortality. This study adds the most definitive evidence to date of an association between recreational physical activity done before lung cancer diagnosis and survival outcomes.

There were 10 previous publications on the association between physical activity done either before or after lung cancer diagnosis and survival after lung cancer (2-11). In our published systematic review and meta-analysis (12) that we have recently updated with 1 additional study, we estimated that prediagnosis physical activity decreased risk of lung cancer-specific mortality by 19% (hazard ratio = 0.81, 95% confidence interval = 0.75 to 0.87) when comparing the highest with the lowest categories of activity level in 6 studies that provided estimates. For all cause-mortality, we estimated, based on 3 studies, there was a 27% decrease (hazard ratio = 0.73, 95% confidence interval = 0.60 to 0.94) with higher vs lower levels of postdiagnosis recreational physical activity. Of note, in the study by Yang and colleagues (1), only prediagnosis activity levels were available. Evidence from our systematic review (12) suggests that postdiagnosis activity levels have a greater impact on reducing both cancer-specific and all-cause mortality risks across cancer sites, including lung cancer. Hence, this evidence supports recommendations for cancer survivors to increase their levels of activity after diagnosis to improve their probability of survival (13).

Several strengths in the pooled analysis conducted by Yang and colleagues (1) need to be highlighted. These investigators were able to combine 11 cohort studies conducted worldwide—7 in the USA, 2 across Europe, and 2 in China—which provides a fairly representative sample of the global population of lung cancer survivors. The sample size was considerable with more than 20 000 cases, of which nearly 17 000 had died, including nearly 14 000 lung cancer deaths; this enabled precise measures of the associations. Furthermore, data were available on tumor stage, grade, and histological type, which permitted a detailed assessment of mortality risk within these case subgroups that was not previously possible. The list of covariates selected a priori included major confounders and prognostic factors for lung cancer survival except for treatment data.

The main limitations of this study (1) are related to the measurement of physical activity. Given the heterogeneity of methods used to assess physical activity across these cohorts, the authors were limited to examining only prediagnosis leisure-time physical activity. Furthermore, participants were categorized into 3 broad categories for these analyses (no activity, low active [>0-8.3 MET-h/wk], and moderately/highly active [≥8.3 MET-h/wk]). The moderately active group achieved the recommended levels for health benefits of 8.3-16.0 MET-h/wk equivalent to 150-300 minutes of moderate or 75-150 minutes of vigorous intensity activity per week. Although this study had a large sample size, too few participants were highly active (>16.0 MET-h/wk); hence, this level of activity could not be assessed separately from those who were moderately active.

Yang et al. (1) have demonstrated the value of combining large cohort studies and conducting individual-level pooled data analyses that overcome many of the limitations of meta-analyses restricted to the published data. The next steps needed to overcome the limitations of their pooled analysis will be prospectively coordinated cohort studies conducted worldwide that include ethnically diverse populations and that use harmonized and standardized measures of physical activity that are a combination of direct measures and self-report questionnaires. These studies will require an extensive examination of confounders, effect modifiers, and a complete assessment of prognostic factors, including cancer treatments received.

A key question that remains unresolved for both lung cancer risk and survival is the role of physical activity in mitigating the impact of smoking behaviors. Tobacco smoking is the most well-established lung cancer risk factor, with an estimated 72% of lung cancers and 17.5% of all cancers being directly attributable to this exposure and an additional 6% of lung cancers and 0.8% of all cancers attributable to passive tobacco smoking exposure (14). The paradox found with lung cancer risk is the apparent protective effect of physical activity apparent among ever smokers but not for nonsmokers (15). Yang and colleagues (1) noted a statistically significant protective effect of physical activity among former smokers, with a 20% reduction in all-cause mortality. For never smokers, a reduction in mortality risk was also observed, albeit not statistically significant and no association with physical activity was found among current smokers. These associations were not clearly observed for lung cancer–specific mortality, and residual confounding by smoking was acknowledged by the authors as a limitation.

Yang and colleagues (1) have provided a clearer understanding of the role of prediagnosis recreational physical activity in improving survival after lung cancer. Although the evidence supporting a protective effect of physical activity on lung cancer risk appears weak (16), engaging in recreational physical activity before cancer diagnosis remains important for favorable survival outcomes among lung cancer survivors. Given the high morbidity and mortality associated with lung cancer, this pooled analysis provides welcome credibility to the rapidly accumulating observational evidence base that physical activity is a safe, effective adjuvant to conventional cancer treatments that can prolong both the quality and quantity of life after a cancer diagnosis. There remains an urgent need to delineate the exact type, dose, and timing of physical activity required to achieve those objectives by cancer site and by clinical-pathologic characteristics of each cancer patient. The ultimate objective is to be able to prescribe exercise interventions to cancer patients that are targeted and appropriate to them, their cancer, and their personal situation and that will improve their outcomes. This objective is rapidly coming closer to reality.

## Funding

None.

## Notes

**Role of the funder:** Not applicable.

**Disclosures:** The authors have no disclosures.

**Author contributions:** CMF, LY: Writing—original draft; writing—review and editing.

CMF, who is a *JNCI Cancer Spectrum* Associate Editor and a co-author on this editorial, was not involved in the editorial review or decision to publish this editorial.

## Data Availability

No new data were generated or used for this editorial. All data cited can be found in the referenced sources.
